# Unique expansion of IL-21+ Tfh and Tph cells under control of ICOS identifies Sjögren’s syndrome with ectopic germinal centres and MALT lymphoma

**DOI:** 10.1136/annrheumdis-2020-217646

**Published:** 2020-09-22

**Authors:** Elena Pontarini, William James Murray-Brown, Cristina Croia, Davide Lucchesi, James Conway, Felice Rivellese, Liliane Fossati-Jimack, Elisa Astorri, Edoardo Prediletto, Elisa Corsiero, Francesca Romana Delvecchio, Rachel Coleby, Eva Gelbhardt, Aurora Bono, Chiara Baldini, Ilaria Puxeddu, Piero Ruscitti, Roberto Giacomelli, Francesca Barone, Benjamin Fisher, Simon J Bowman, Serena Colafrancesco, Roberta Priori, Nurhan Sutcliffe, Stephen Challacombe, Gianluca Carlesso, Anwar Tappuni, Costantino Pitzalis, Michele Bombardieri

**Affiliations:** 1 Centre for Experimental Medicine and Rheumatology, William Harvey Research Institute, London, England, UK; 2 Immuno-Allergology Unit, Department of Clinical and Experimental Medicine, University of Pisa, Pisa, Italy; 3 Oncology R&D, Astrazeneca, Gaithersburg, Maryland, USA; 4 University of Pisa, Rheumatology Unit, Pisa, PI, Italy; 5 Department of Clinical Sciences and Applied Biotechnology, University of L'Aquila, L'Aquila, Abruzzo, Italy; 6 RRG, Institute of Inflamation and Ageing, University of Birmingham, Birmingham, UK, UK; 7 NIHR Birmingham Biomedical Research Centre, University Hospitals Birmingham NHS Foundation Trust, Birmingham, UK; 8 Dipartimento di Medicina Interna e Specilità Mediche, UOC Reumatologia, Universita degli Studi di Roma La Sapienza Facolta di Medicina e Odontoiatria, Roma, Lazio, Italy; 9 Rheumatology, Barts Health NHS Trust, London, London, UK; 10 Oral Medicine, KCL Dental Institute, London, UK; 11 Early ICA Discovery, Early Oncology R&D, AstraZeneca, Gaithersburg, Maryland, USA; 12 Institute of Dentistry, Barts and The London School of Medicine and Dentistry, London, London, UK

**Keywords:** sjogren's syndrome, t-lymphocyte subsets, cytokines, autoimmune diseases

## Abstract

**Objectives:**

To explore the relevance of T-follicular-helper (Tfh) and pathogenic peripheral-helper T-cells (Tph) in promoting ectopic lymphoid structures (ELS) and B-cell mucosa-associated lymphoid tissue (MALT) lymphomas (MALT-L) in Sjögren’s syndrome (SS) patients.

**Methods:**

Salivary gland (SG) biopsies with matched peripheral blood were collected from four centres across the European Union. Transcriptomic (microarray and quantitative PCR) analysis, FACS T-cell immunophenotyping with intracellular cytokine detection, multicolor immune-fluorescence microscopy and *in situ* hybridisation were performed to characterise lesional and circulating Tfh and Tph-cells. SG-organ cultures were used to investigate functionally the blockade of T-cell costimulatory pathways on key proinflammatory cytokine production.

**Results:**

Transcriptomic analysis in SG identified Tfh-signature, interleukin-21 (IL-21) and the inducible T-cell co-stimulator (ICOS) costimulatory pathway as the most upregulated genes in ELS+SS patients, with parotid MALT-L displaying a 400-folds increase in IL-21 mRNA. Peripheral CD4^+^CXC-motif chemokine receptor 5 (CXCR5)^+^programmed cell death protein 1 (PD1)^+^ICOS^+^ Tfh-like cells were significantly expanded in ELS+SS patients, were the main producers of IL-21, and closely correlated with circulating IgG and reduced complement C4. In the SG, lesional CD4^+^CD45RO^+^ICOS^+^PD1^+^ cells selectively infiltrated ELS+ tissues and were aberrantly expanded in parotid MALT-L. In ELS+SG and MALT-L parotids, conventional CXCR5^+^CD4^+^PD1^+^ICOS^+^Foxp3^-^ Tfh-cells and a uniquely expanded population of CXCR5^-^CD4^+^PD1^hi^ICOS^+^Foxp3^-^ Tph-cells displayed frequent IL-21/interferon-γ double-production but poor IL-17 expression. Finally, ICOS blockade in *ex vivo* SG-organ cultures significantly reduced the production of IL-21 and inflammatory cytokines IL-6, IL-8 and tumour necrosis factor-α (TNF-α).

**Conclusions:**

Overall, these findings highlight Tfh and Tph-cells, IL-21 and the ICOS costimulatory pathway as key pathogenic players in SS immunopathology and exploitable therapeutic targets in SS.

**Key messages**

**What is already known about this subject?**

- In Sjogren’s syndrome (SS), germinal centres (GC) forming ectopically in salivary glands (SG) function as niches for autoreactive B-cells, which formation believed to results from the GC B and T-cell interaction. Ectopic GC are associated with evolution to B cell mucosa-associated lymphoid tissue (MALT) lymphoma in several studies.
- T-follicular-helper (Tfh) and recently described pathogenic peripheral-helper T-cells (Tph) are key mediators in (autoreactive) B-cell differentiation through inducible T-cell costimulator (ICOS)-ICOS-L interaction and interleukin-21 (IL-21) production, but their relevance has not been investigated in SS ectopic GC reaction and MALT lymphoma.

**What does this study add?**

- Tfh and Tph-cells are enriched in both SS peripheral blood and SG with GC, invariably express ICOS, represent the main source of IL-21 and frequently coexpress IL-21/interferon-γ, especially in parotid MALT-lymphoma.
- ICOS blockade in *ex vivo* SG-organ cultures significantly reduced the production of IL-21 and inflammatory cytokines IL-6, IL-8 and tumour necrosis factor-α (TNF-α).

**How might this impact on clinical practice or future developments?**

- Tfh and Tph-cells, IL-21 and the ICOS costimulatory pathway can be considered biomarkers of ectopic GC, may be used for patient stratification and represent exploitable therapeutic targets in patients with SS.

## Introduction

Sjogren’s syndrome (SS) is characterised by lymphocytic infiltration of the exocrine glands, mainly the lacrimal and salivary glands (SG).^1^ The pathogenic role of B-cells in SS is a hallmark of the disease including the presence of circulating autoantibodies, alterations in peripheral B-cell subpopulations,^2^ B-cell predominance in advanced SG lesions^3^ and the increased risk of developing non-Hodgkin B-cell mucosa-associated lymphoid tissue (MALT)-lymphoma (MALT-L) in SS.^4^ In around 30%–40% of patients with SS, B-cell infiltrates forming in minor (labial) SG are organised in ectopic germinal centres (GC)^5^; follicles formed by aggregates of segregated B and T-cells endowed with a follicular dendritic cell (FDC) network. These structures, also known as ectopic lymphoid structures (ELS), function as niches for autoreactive B-cells.^6^

In physiological GC responses, efficient T-cell-dependent antigen-driven B-cell response *in vivo* depends on the development of functional GCs which require T-follicular helper (Tfh) cells, 7–9 where Tfh-secreted interleukin-21 (IL-21) is a critical factor for B-cell maturation.^7 10–12^ Tfh-cells are highly specialised CD4^+^ memory T-helper cells, characterised by high expression of the CXC-motif chemokine receptor 5 (CXCR5), the inducible T-cell costimulator (ICOS) molecule, the coinhibitory molecule programmed cell death protein 1 (PD-1) and the transcription factor Bcl6.^13^ Tfh-cells migrate to the B-cell follicle in response to the FDC- produced CXCR5 ligand, CXCL13.^14^ At the border with and inside the GC, Tfh-cells interact with B-cells through ICOS and its ligand, ICOSL, releasing high amounts of IL-21.^15 16^ Given their fundamental role as mediators of B-cell activation and antibody production, it is not surprising that Tfh-cells together with IL-21 have been linked to autoimmune diseases characterised by a B-cell hyperactivation and dysregulated GC response, including SS.^17 18^ Interestingly, recent work described alternative IL-21-producing Tfh-like cells (also designated ‘pathogenic T peripheral helper cells (Tph)’) as able to localise at inflammatory sites, such as RA synovium, in the absence of CXCR5 expression.^19^ Tph cells lack prototypic Tfh markers like CXCR5 and Bcl6 but expresses high levels of IL-21 and CD40L. Similarly to canonical Tfh, Tph-cells isolated from inflamed tissue^19 20^ can drive the differentiation of B-cells into antibody-secreting cells *in vitro*.^19^

To date, the relevance of Tfh and Tph-cells in the development of SG ELS and evolution to MALT-L in SS has not been clarified. To address this question, we performed a comprehensive investigation in matched SG histology, transcriptomic and lesional/peripheral T-cell immunophenotyping validated in samples from four different centres which identified a subset of Tfh and Tph-cells expressing high levels of IL-21 and/or IL-21/interferon-γ (IFN-γ) under the control of ICOS as a uniquely expanded population able to identify patients with SS with ELS and MALT-L.

## Materials and methods

### Patients sample collection

Blood and labial SG biopsies were collected after informed consent from patients with SS (n=83) fulfilling the 2002 revised criteria of the American-European Consensus Group,^21^ with non-specific chronic sialadenitis (NSCS) (n=65) and from healthy donors (HD) (n=12). SS parotids (n=15) with low-grade MALT-L and parotid adenocarcinoma (n=10) were obtained from the Guy’s Hospital Oral Pathology. MALT-L was diagnosed histologically by the presence of halos of monocytoid B-cells infiltrating epimyoepithelial islands and by variable, diversity and joining (VDJ) genes PCR for the heavy chain Ig genes^22^ for clonal populations.

### Patient and public involvement

There were no funds or time allocated for patient and public involvement so we were unable to involve patients. Patients were invited to help us developing our dissemination strategy.

### Immunohistochemistry and immunofluorescence) on SG tissue

After deparaffinisation, formalin-fixed paraffin-embedded sections were incubated with antigen retrieval solution pH6 (Dako) in pressure cooker (for CD20, CD138, CD3, CD68, CD4, CD45RO, PD1, ICOS, BCL6) or with proteinase-K (Dako) (for CD21). After appropriate blocking steps and primary antibody (online supplementary table S1) staining, slides were incubated with HRP-conjugated secondary antibody and developed with DAB (Dako). For immunofluorescence (IF), slides were incubated with the relevant secondary antibodies (online supplementary table S1) prior to 4′,6-diamidino-2-phenylindole (DAPI) nuclear counterstaining. Images were captured using Olympus BX61 Motorised Microscope or confocal microscope (Leica DM5500Q).

10.1136/annrheumdis-2020-217646.supp1 Supplementary data

### Flow cytometry

Flow-cytometry analysis was performed on 52 frozen peripheral blood mononuclear cells (PBMCs) prior thawing. Egressed cells from one parotid MALT-L and PBMCs were stimulated with PMA (50 ng/mL), ionomycyn (750 ng/mL), Brefeldin-A (10 µg/mL) in RPMI complete (RPMI +10% fetal bovine serum) medium, for 3 hours. Cells were stained using Zombie Aqua Live/Dead kit (Biolegend) for 15 min, washed and incubated for 10 min with human Fc TruStain FcX (Biolegend). Cells were stained for surface antigens, fixed, permeabilised (fixation-permeabilisation buffer; eBioscience) and stained for intracellular cytokines. Antibodies used are listed in online supplementary table S2. Cells were acquired using a LSR Fortessa II (BD Biosciences) flow cytometer and analysed with FlowJo V.10 software.

### Whole-genome microarray analysis and quantitative gene expression profiling in human SG tissue

For whole-genome microarray, biotin-labelled amplified complementary RNA (cRNA) from total SG RNA was amplified according to the MessageAmp Premier Protocol (ThermoFisher). cRNA (20 µg) was fragmented for hybridisation on Affymetrix Human Genome U133 Plus 2.0 GeneChip arrays. Data capture and quality assessments were performed with the GeneChip Operating Software tool and data analysis with R using affy, frma and dependent packages to generate a scaled gene expression matrix. eBayes R package was used for statistical analysis of differential transcript expression and gene signature analysis was performed using the *GSVA* package (Gene Set Variation Analysis)^23^ and a collection of established gene signatures. A list of genes defining the gene signatures are listed in online supplementary table S3. Targeted quantitative TaqMan RT-PCR was performed as previously described^24^; primers and probes are listed in online supplementary table S4.

### RNAScope fluorescence *in situ* hybridisation for IL-21 RNA

*In situ* hybridisation for IL-21 RNA (NM_021803.3) was performed following manufacturer’s instructions (Advanced Cell Diagnostics) on optimally prepared paraffin SG tissue. RNAscope probe Hs-IL21 (code 401251) was applied for 2 hours at 40°C and the signal was detected using Alexa Fluor 488 Tyramide Reagent (ThermoFisher, code B40953). Slides were then washed in PBS and stained for ICOS (Abcam, code Ab105227) and CD4 (Dako, code M7310) overnight. After incubation with secondary antibodies, slides were DAPI counterstained and mounted in ProLong Gold (ThermoFisher, code P36930). Slides were digitised using NanoZoomer S60 slide scanner (Hamamatsu Photonics). DapB (code 310043) and Hs-POLR2A (code 310451) were used as negative and positive control probes, respectively.

### ICOS blockade *in vitro* assay

SG-organ cultures were performed in complete RPMI medium. Each SG lobule was cut in half and each half incubated with either anti-ICOS blocking antibody (clone JTA-009)^25 26^ or its isotype control (10 µg/mL), for 3 days and MALT-L for 24 hours, due to the different time for cell egression. Multiple samples from one parotid MALT-L parotidectomy and 3 SS patient labial SG (2–8 lobules per patient) were tested. All supernatants were collected and frozen before analysis.

### Protein detection in sera and supernatants

IL-21 levels in serum was quantified using an IL-21 ELISA (Biolegend, code 433804) following manufacturer’s instructions. In the organ culture supernatant, cytokines levels were screened with Proteome profiler human XL cytokine array kit (R&D, code ARY022B) in line with manufacturer’s instruction. Cytokines were quantified using customised multiplex liquid phase immunoassay (Biolegend, LegendPlex) and/or with specific ELISA assays (IL-6 and IL-8, Biolegend, 430 504 and 431 504, respectively).

### Statistical analysis

Differences in quantitative variables were analysed by the Mann-Whitney U-test when comparing two groups and by Kruskal-Wallis with Dunn’s post-test correction when comparing multiple groups. χ^2^ test with Yates’ correction when required or Fisher’s exact test when appropriate were used to evaluate associations of qualitative variables in the different groups. Spearman’s rank analysis was performed for non-parametric variable correlations. All the statistical analyses were performed using GraphPad Prism V.7 for Windows (GraphPad Software, USA).

## Results

### Expansion of circulating IL-21 producing Tfh cells identifies patients with SS with systemic immune activation and higher SG infiltration

We first analysed circulating IL-21 level and the frequency of IL-21 production in conventional Tfh (CXCR5^+^PD1^+^ICOS^+^) and the recently described Tph (CXCR5^-^PD1^hi^ ICOS^+^) in patients with SS and their correlation with clinical, immunological and histopathological activity. Patient characteristics are summarised in table 1.

**Table 1:** Summary of the patient characteristics

Complete cohort	Sjögren’s syndrome n=83	NSCS n=65	Healthy donors n=12
Gender, F/M	75/8	57/8	11/1
Age in years mean	54 [55] (14.0)	55 [56] (13.3)	43 [44] (7.4)
[median] (SD)			
Primary/ Secondary Sjogren’s syndrome	74/9		
Disease duration in years	5.9 [3] (6.0)	5.1 [3] (5.6)	
Mean [median] (SD)			
ESSDAI	5.5 [5] (4.9)	n/a	
Mean [median] (SD)			
Anti-Ro	59%	0%	
Positive of total			
Anti-La	35%	0%	
Positive of total			
Rheumatoid factor	51%	11%	
Positive of total			
Serum IgG (g/L)	15.4 [14.0] (5.8)	11.0 [11.1] (3.7)	
Mean [median] (SD)			
Serum IgA (g/L)	2.9 [2.3] (1.8)	2.8 [2.5] (2.4)	
Mean [median] (SD)			
Serum IgM (g/L)	1.5 [1.1] (2.0)	1.3 [1.1] (0.7)	
Mean [median] (SD)			
Serum C3 (g/L)	1.2 [1.2] (0.3)	1.5 [1.3] (1.3)	
Mean [median] (SD)			
Serum C4 (g/L)	0.2 [0.2] (0.1)	0.3 [0.3] (0.1)	
Mean [median] (SD)			
Lymphocytes count (x10^9^/L)	1.6 [1.5] (0.6)	2.2 [2.1] (0.8)	
Mean [median] (SD)			
Treatments			
Hydroxychloroquine csDMARDs*	35/83 8/83		
Steroids	5/83		

*csDMARDs other than hydroxychloroquine.

csDMARDs, conventional synthetic disease-modifying antirheumatic drugs; ESSDAI, EULAR Sjogren's syndrome disease activity index; NSCA, non-specific chronic sialadenitis.

**Figure 1:**
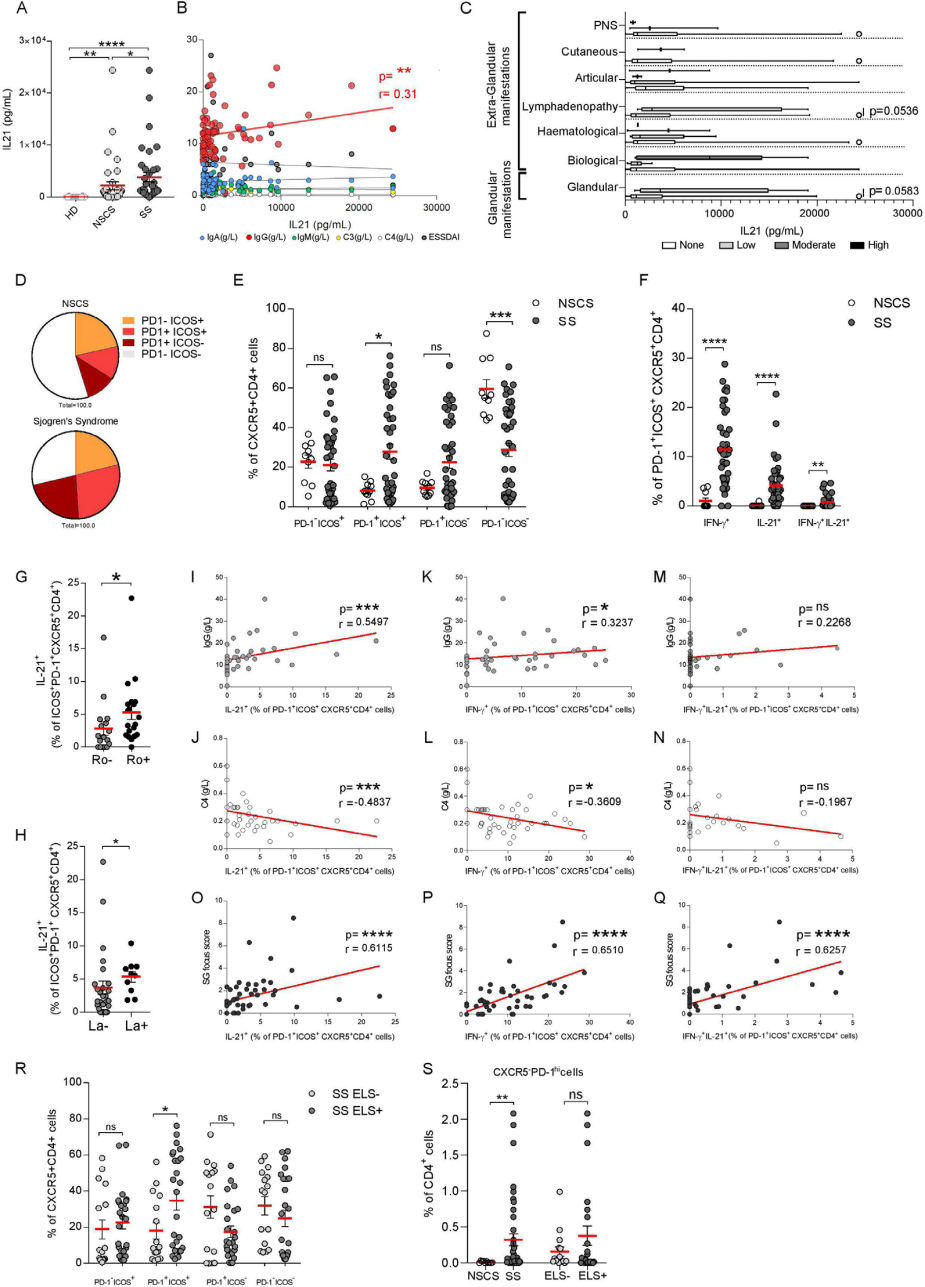
IL-21 and T follicular helper (Tfh) cells in peripheral blood of SS and NSCS patients. (A) ELISA quantification of IL-21 serum level (pg/mL) from HD (n=12), NSCS (n=37) and SS (n=37). (B) Correlations of IL-21 serum level (pg/mL) with serum level of IgG, IgM, IgA, C3, C4 and ESSDAI in NSCS (n=37) and SS (n=37) patients. Spearman and p value are shown for the significant correlation (IgG, in red). (C) IL-21 serum level (pg/mL) in SS (n=31) according to ESSDAI domains. Box and whiskers plot show median and 5–95 percentile. Statistical analysis by Kruskal-Wallis-test with Dunn’s post-test correction for multiple comparison (A). (D) Average frequencies of Tfh-cell subsets, identified on the basis of ICOS and PD-1 expression and their frequencies distribution (E) in NSCS (white dots, n=10) and SS (dark grey dots, n=42). (F) Frequency of IFN-γ^+^, IL-21^+^ and IFN-γ^+^-IL-21^+^ double producing cells as percentage of PD-1^+^ICOS^+^, gated on CXCR5^+^CD4^+^ cells, detected by flow-cytometry on PBMC stimulation with PMA and ionomycin. Frequency of IL-21^+^ cells segregating the SS cohort for Ro (G) (Ro- (n=17) and Ro+ (n=20)) and La presence (H) (La- (n=27) and La+ (n=10)). Spearman correlation of IL-21^+^ cell (I, J, O), IFN-γ^+^ (K, L, P) or IL-21^+^-IFN-γ^+^ cell frequency (M, N, Q) with IgG (g/L) and C4 (g/L) serum levels and SG focus score in NSCS (n=10) and SS (n=42) patients. (R) Frequency of Tfh-cells subsets identified on the basis of ICOS and PD-1 expression in SS cohort segregated for ELS presence (ELS-, light grey dots (n=17), ELS+, dark grey dots (n=25)). (S) Frequency of CXCR5^-^PD-1^hi^ cells, as percentage of CD4^+^ cells, in NSCS (n=10) and SS (n=42), and segregating the SS cohort for the presence of ELS. Statistical analysis by Mann-Whitney U t-test in (C), (E), (F), (G), (H), (R), (S). All graphs represent mean±SEM. *p<0.05, **p<0.01, ***p<0.001, ****p<0.0001. ELS, ectopic lymphoid structure; HD, healthy donor; ICOS, inducible T-cell costimulator; IFN-γ, interferon-γ; IL-21, interleukin-21; NSCS, non-specific chronic sialoadenitis; PD-1, programmed cell death protein 1; PNS, peripheral nervous system; SS, Sjogren’s syndrome.

IL-21 serum levels were increased in patients with SS in comparison to NSCS patients and HD (figure 1A), but showed no correlation with ESSDAI or immunological abnormalities (figure 1B). Also, IL-21 levels did not differ between patients with SS stratified for extraglandular involvement (figure 1C) or ELS (online supplementary figure S1) in the SG (online supplementary figure S2A).

Because of the heterogeneity of markers used so far to define Tfh-cells,^18^ we performed a comprehensive analysis of Tfh-cell subsets, by using PD-1 and ICOS expression to identify *bona-fide* Tfh-cells as CD4^+^CD25^-^Foxp3^-^CXCR5^+^ICOS^+^PD-1^+^,^27^ assessing also their functional capacity to produce IFN-γ, IL-17 and IL-21 (online supplementary figure S2B).

Circulating CXCR5^+^CD4^+^ T-cells were higher in patients with SS compared with NSCS (online supplementary figure S2C), with no differences in the general CD4^+^ frequency (online supplementary figure S2D). More specifically, patients with SS had an expansion of circulating CXCR5^+^ICOS^+^PD-1^+^ activated Tfh-cells (figure 1D, E, online supplementary figure S2E, O) which displayed significantly increased IFN-γ, IL-21 and double IL-21/IFN-γ production in patients with SS compared with NSCS (figure 1F). Circulating IL-21-producing CXCR5^+^ICOS^+^PD-1^+^ (figure 1G, H) but not total CXCR5^+^ICOS^+^PD-1^+^ or IFN-γ producing Tfh-cells (online supplementary figure S2F–H) were higher in patients with SS with anti-Ro/SSA and anti-La/SSB autoantibodies. Moreover, circulating IFN-γ^+^ and IL-21^+^CXCR5^+^ICOS^+^PD-1^+^ Tfh-cells positively correlated with serum IgG levels and inversely with complement C4 (figure 1I–L) unlike IL-21/IFN-γ double producers (figure 1M, N) and the total CXCR5^+^ICOS^+^PD-1^+^ Tfh subset (online supplementary figure S2I–J).

Next, we investigated whether circulating Tfh-cells could identify patients with SS with more extensive SG infiltration and the presence of SG ELS (online supplementary figure S1). IL-21, IFN-γ and IL-21/IFN-γ-producing CXCR5^+^ICOS^+^PD-1^+^ Tfh-cells (figure 1O–Q), but also total CXCR5^+^ICOS^+^PD-1^+^ Tfh-cells (online supplementary figure S2K) closely correlated with the SG focus score while circulating CXCR5^+^CD4^+^ T-cells double expressing ICOS^+^PD-1^+^ were higher in patients with SS with ELS (figure 1R and online supplementary figure 2L, M, P).

We next analysed the recently described population of Tph cells^19^ defined as CXCR5^-^PD1^hi^CD4^+^ T-cells. Although circulating Tph were increased in the blood of patients with SS compared with NSCS, as previously described,^28^ we did not observe a selective expansion of Tph in the ELS+ subgroup (online supplementary figure 1S).

### Transcriptomic analysis of Tfh-cell signature and an increased IL-21 and IL-21R mRNA expression identify SG tissue with ELS

**Figure 2:**
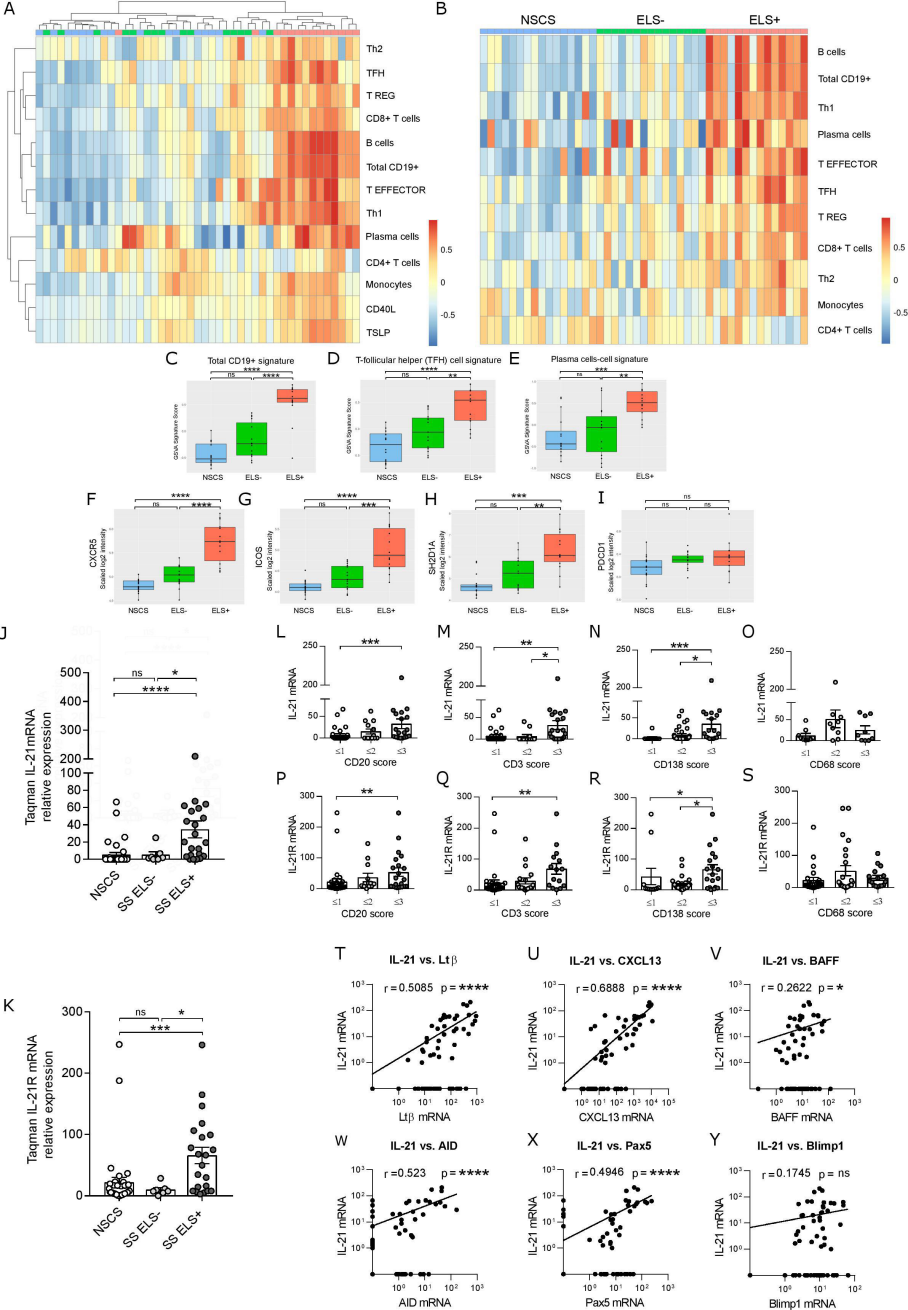
Tfh-cell signature and IL-21 expression correlate with lymphocytic infiltration, ELS formation and GC B-cell genes in SS SGs. (A) Unsupervised whole-genome microarray analysis from SG RNA of NSCS (n=15) and SS (n=30), segregated for the absence (ELS-, n=15) and the presence of ELS (ELS+, n=15). (B) Supervised whole-genome microarray analysis of cohort described in (A), with the signature pathways rank-ordered for expression intensity. (C–E) GSVA for indicated signatures and (F–I) gene expression intensity comparison of Tfh-cell signature pathway genes (encoding respectively for CXCR5, ICOS, SAP and PD-1) in NSCS (n=15), ELS- (n=15) and ELS+ (n=15) SG. Linear regression model statistics. All graphs represent mean±SEM. *P<0.05, **P<0.01, ***P<0.001, ****P<0.0001. Real-time PCR expression for IL-21 (J) and IL-21 receptor (IL21R) (K) on total RNA from SG biopsies from NSCS (n=37) and SS (n=29) patients, segregated on the basis of absence (ELS-, n=7) and presence of ELS (ELS+, n=22). Quantification of IL-21 mRNA (L–O) and IL-21R expression (P–S) in SG tissue biopsies, according to histological semi-quantitative score (0 to 3) of B (CD20), T (CD3), plasma cells (CD138) and macrophages (CD68). Statistical analysis by Kruskal-Wallis-test with Dunn’s post-test correction for multiple comparison. (T–Y) Spearman correlation analysis between IL-21 mRNA in SG and indicated lymphoid and GC B-cell genes. All graphs represent mean±SEM. *p<0.05, **p<0.01, ***p<0.001, ****p<0.0001. CXCR5, CXC-motif chemokine receptor 5; ELS, ectopic lymphoid structure; GC, germinal centres; GSVA, Gene Set Variation Analysis; ICOS, inducible T-cell co-stimulator; IL-21, interleukin-21; NSCS, non-specific chronic sialoadenitis; PD-1, programmed cell death protein 1; SAP, SLAM-associated protein; SG, Salivary gland; SS, Sjogren’s syndrome; Tfh, T-follicular-helper.

We next performed transcriptomic analysis of SG tissue comparing ELS+ SS, ELS- SS and NSCS patients. Unsupervised clustering showed that ELS+ patients clustered differently from ELS- and NSCS (figure 2A). A supervised analysis, by pre-classifying patients into ELS+, ELS- and NSCS, gave similar results, showing a clear association between B-cells, plasma cells and Tfh signatures with the presence of ELS in SG (figure 2B). GSVA score confirmed that B-cell, Tfh-cell and plasma-cell signatures all segregate ELS+ from ELS- and NCSC patients (figure 2C–E). On analysing the Tfh signature, we identified 4 Tfh-cell associated genes: CXCR5, ICOS, PDCD1, SH2D1A as most upregulated in ELS+SS patients (figure 2F–I). SH2D1A gene encodes for SLAM-associated protein that stabilises B and T-cell interaction and is essential for the differentiation of functional Tfh-cells.^29^

Although the IL-21/IL-21R pathway has been implicated in the pathogenesis of SS,^30^ there is no available evidence of its implication in ELS development. Thus, we next performed a targeted gene expression profiling of IL-21 and IL-21R together with other ELS and GC B-cell-related genes and showed that IL-21 and IL-21R mRNA were significantly increased in the SG of ELS+SS compared with ELS- SS and NSCS patients (figure 2J, K). Additionally, IL-21/IL-21R gene expression levels increased in parallel with the infiltrating CD20^+^ B-cells, CD3^+^ T-cells and CD138^+^ plasma cells, but not CD68^+^ macrophages as assessed by immunohistochemistry (figure 2L–S). Furthermore, IL-21 mRNA also correlated with genes associated with ELS (LTB, CXCL13) and functional B-cell activation (BAFF, AICDA, PAX5) (figure 2T–Y). Overall, these data suggest a role for the IL-21/IL-21R pathway in driving local B-cell activation within ELS in SG.

### PD1^+^ICOS^+^CD4^+^ T-cells infiltrate ELS+ SG, express IL-21 and co-localise with ectopic B-cell follicles

**Figure 3:**
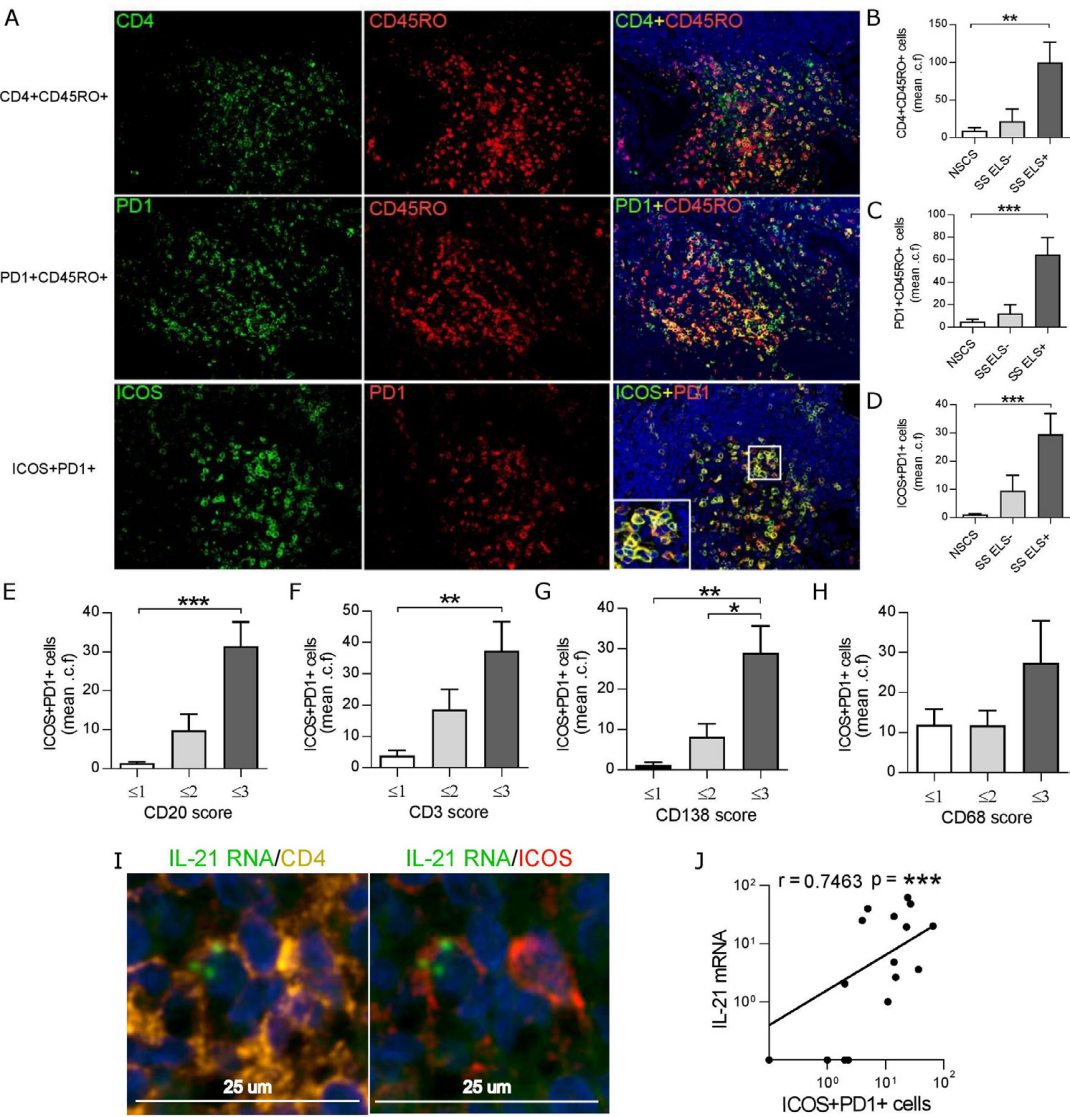
PD-1^+^ICOS^+^CD45RO^+^CD4^+^ cells are increased within SG with ELS in SS and produce IL-21. (A) Representative immunofluorescence detection of CD4^+^CD45RO^+^ (top row), PD1^+^CD45RO^+^ (middle row) and ICOS^+^PD1^+^ cells (bottom row) in SG biopsy tissues with ELS. (B–D) Quantification (mean counts per field, a minimum of 5 random fields) of the double positive cells for each double immunofluorescence combination in SG biopsy tissues from NSCS (n=8) and SS (n=12) patients. Images displayed at x20 magnification. (E–H) ICOS^+^PD1^+^ cell count was segregated according to histological semi-quantitative score (0 to 3) of B (CD20), T (CD3) plasma cells (CD138) and macrophages (CD68). Statistical analysis by Kruskal-Wallis-test with Dunn’s post-test correction for multiple comparison (B–H). (I) Representative fluorescent in situ hybridisation (FISH) detection of IL-21 RNA, costained with CD4 and ICOS in SG biopsy tissues with ELS. (J) Spearman correlation analysis between SG real-time PCR IL-21 mRNA expression with ICOS^+^PD1^+^ cell count. All graphs represent mean±SEM. *p<0.05, **p<0.01, ***p<0.001. ELS, ectopic lymphoid structure; ICOS, inducible T-cell co-stimulator; NSCS, non-specific chronic sialoadenitis; PD-1, programmed cell death protein 1; SG, salivary gland; SS, Sjogren’s syndrome.

Sequential double IF stainings for memory T-cells (CD4^+^CD45RO^+^), activated-memory T-cells (CD45RO^+^PD1^+^) and Tfh-like cells (PD1^+^ICOS^+^) in SS SGs demonstrated a progressive increase in all cell types, but particularly an enrichment in Tfh-like cells within B-cell follicles in ELS+ (figure 3A–D). Additionally, semiquantitative assessment of cell infiltration demonstrated higher levels of PD1^+^ICOS^+^ cells in patients with high infiltration of B-cells (CD20^+^), T-cells (CD3^+^) and plasma cells (CD138^+^), but not macrophages (CD68^+^) (figure 3E–H). *In situ* hybridisation for IL-21 mRNA in combination with CD4/ICOS immunostaining confirmed that IL-21 expression was confined to ICOS^+^CD4^+^ T-cells within ELS (figure 3I and online supplementary figure S4). In keeping with this data, infiltrating PD1^+^ICOS^+^ T-cell number positively correlated with IL-21 mRNA expression in SS SG (figure 3J).

### Aberrant expansion of IL-21 and ICOS^+^PD1^+^BCL6^+^ infiltrating T-cells in the evolution to parotid MALT B-cell lymphomas

**Figure 4:**
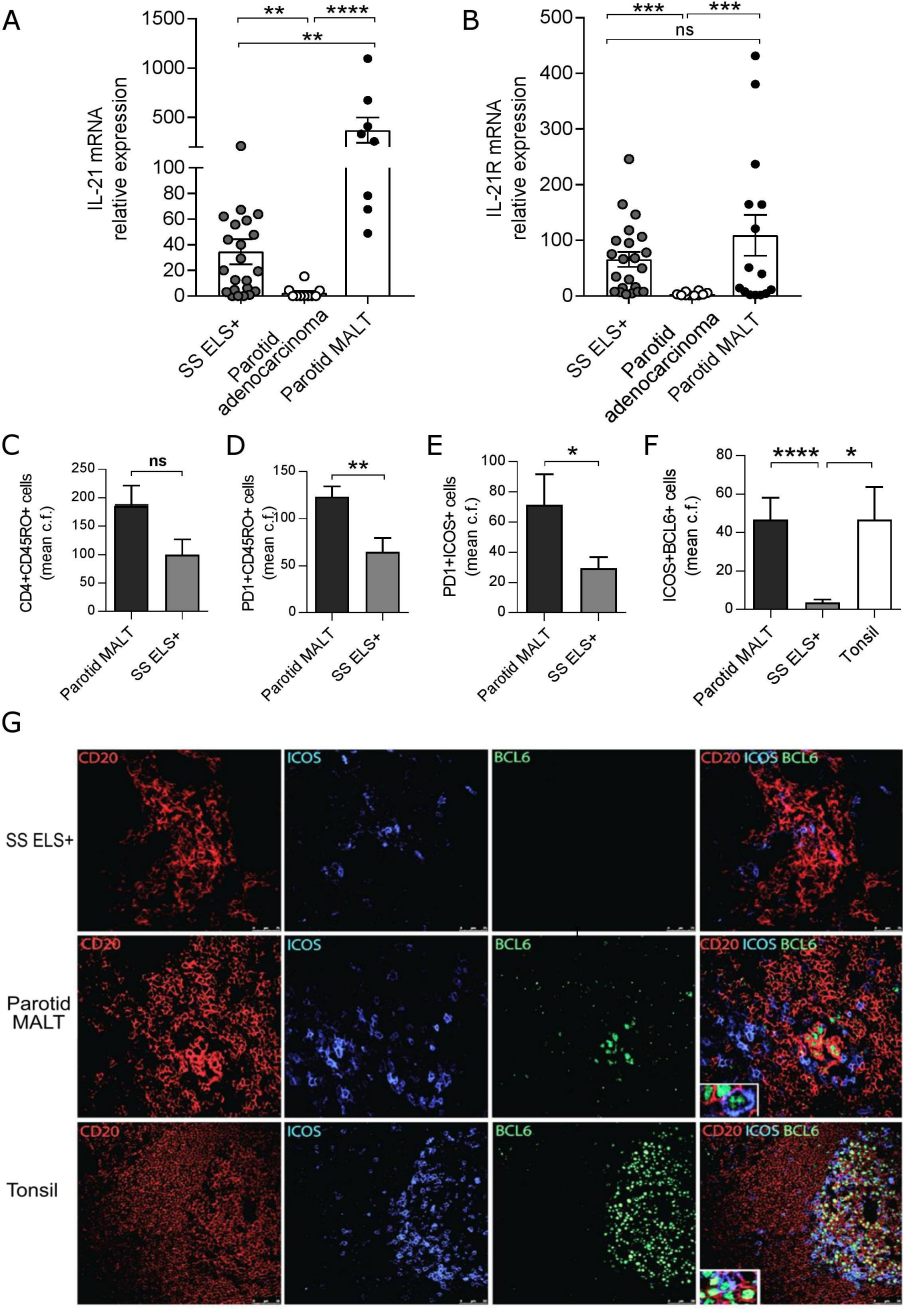
Development of parotid malt lymphomas is associated with elevated SG IL-21 and IL-21R expression. real-time PCR expression for IL-21 (A) and IL21R (B) on RNA extracted from SS labial SG biopsies with ELS (ELS+, n=22), SS parotid SG MALT-lymphoma (n=15) and parotid adenocarcinoma (n=10). Quantification (mean counts per field) of (C) CD4^+^CD45RO^+^, (D) PD1^+^CD45RO^+^, (E) PD1^+^ICOS^+^ cells in SS labial SG biopsies with ELS (ELS+, n=10) and SS parotid SG MALT-lymphoma (n=7). Mann-Whitney U t-test statistics. (F) Mean counts per field of ICOS^+^BCL6^+^ cells between SS parotid SG MALT-lymphoma (n=23), SS labial SG biopsies with ELS (ELS+, n=21) and tonsils (n=3). Statistical analysis by Kruskal-Wallis-test with Dunn’s post-test correction for multiple comparisons (A, B, F). (G) Representative immunofluorescence detection of ICOS^+^BCL6^+^ cells relative to B (CD20+) cell aggregates in SG biopsy tissues with ELS, parotid SG MALT-lymphoma and tonsil. All graphs represent mean±SEM. *p<0.05, **p<0.01, ***p<0.001, ****p<0.0001. ELS, ectopic lymphoid structures; ICOS, inducible T-cell costimulator; IL-21, interleukin-21; MALT, mucosa-associated lymphoid tissue; SG, Salivary gland; SS, Sjögren’s syndrome.

Uncontrolled B-cell activation and aberrant somatic hypermutation^22^ within SS SG can lead to genetic instability and progression to parotid B-cell MALT-L in around 5% of patients with SS,^4^ suggesting that lymphomagenesis in SS is partially sustained by T-cell-driven responses, similarly to gastric MALT-L,^31 32^ although also extra follicular and T cell-independent mechanisms are key in neoplastic B cell expansion.^4^ Therefore, we assessed IL-21 production and Tfh-cells infiltration in SS parotids with MALT-L. First, IL-21 mRNA expression displayed ~400-fold increase in parotid MALT-L compared with control parotids and ~20-fold increase compared with ELS+ minor SG biopsies (figure 4A, B). MALT-L also showed an expanded infiltration of activated-memory (CD45RO^+^PD1^+^) and Tfh-like T-cells (PD1^+^ICOS^+^) to levels significantly higher compared with ELS+SS SGs (figure 4C–E).

Of relevance, within parotid MALT-L, ICOS^+^PD1^+^ T-cells acquired BCL6, a transcription factor essential for Tfh-cell differentiation more frequently than in ELS+ minor SG biopsies (figure 4F) and resided in close proximity to CD20^+^BCL6^+^ GC B-cells (figure 4G).

### Unique SG expansion of Tfh and Tph double IL-21/IFN-γ producers marks the evolution to ELS and parotid B-cell MALT-L

**Figure 5:**
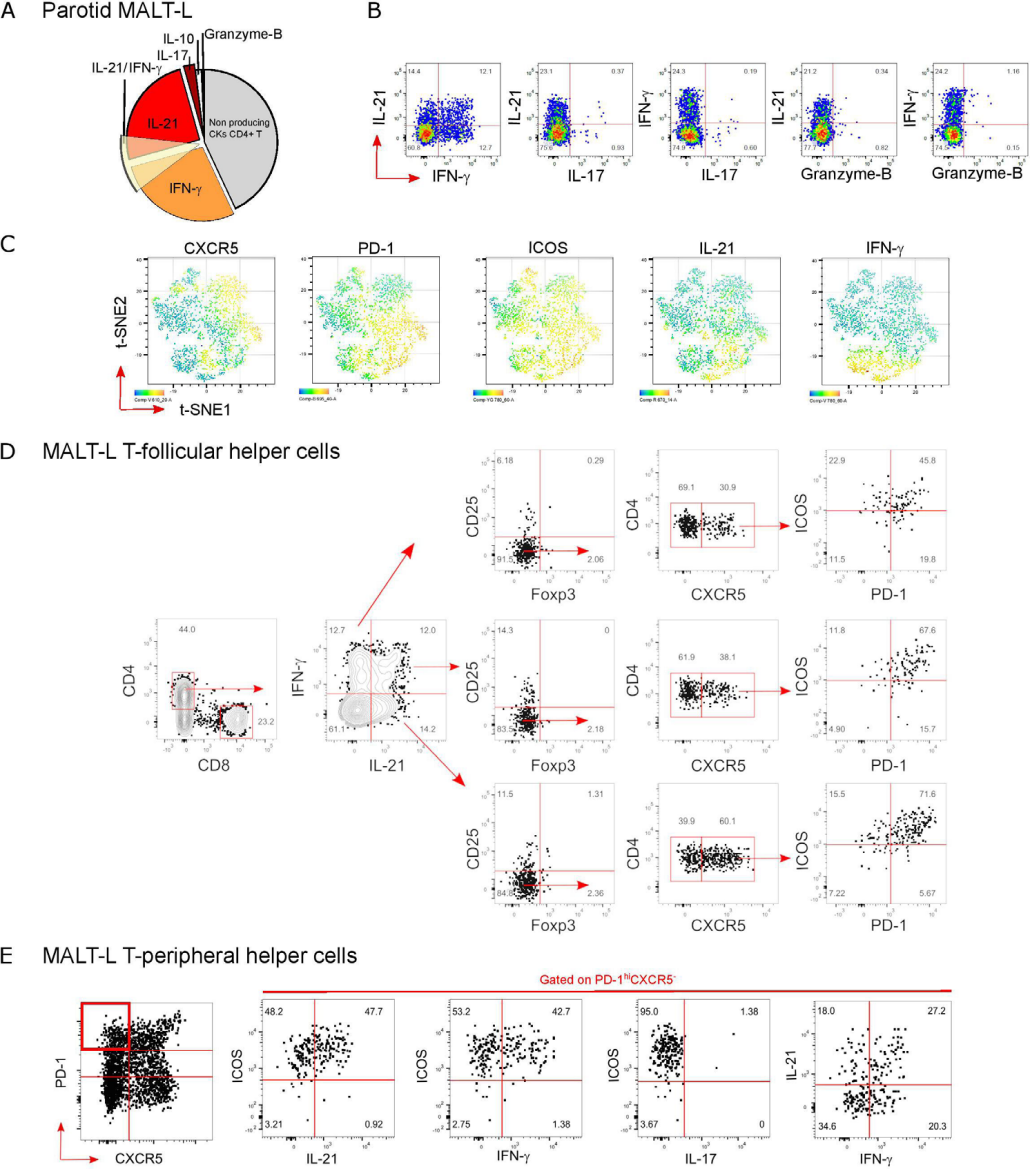
SG inflammation and development of parotid MALT lymphomas is associated with increased Tfh-cell numbers with increased production of IL-21 and IFN-γ. (A) Pie chart showing cytokines production by T helper (CD4^+^) cells isolated from parotid SG MALT-lymphoma (n=1), on stimulation with PMA and ionomycin and flow-cytometry analysis. (B) Representative flow-cytometry dot plots for cytokines production by T helper (CD4^+^) cells and (C) tSNE plots for indicated markers in T helper (CD4^+^) cells from parotid SG MALT-lymphoma. Flow-cytometry gating strategy for the identification of (D) IL-21 and IFN-γ producing Tfh-cells (identified as CD4^+^CD25^-^Foxp3^-^ CXCR5^+^ICOS^+^PD-1^+^) and (E) pathogenic T peripheral helper cells (identified as CD4^+^CD25^-^Foxp3^-^CXCR5^-^ICOS^+^PD-1^+^) in parotid SG MALT-lymphoma (n=1). ELS, ectopic lymphoid structure; ICOS, inducible T-cell costimulator; IFN-γ, interferon-γ; IL-21, interleukin-21; MALT, mucosa-associated lymphoid tissue; NSCS, non-specific chronic sialoadenitis; PD-1, programmed cell death protein 1; SG, Salivary gland; SS, Sjogren’s syndrome; Tfh, T-follicular-helper.

Fluorescence-activated cell sorting (FACS) profiling of T-cell subsets in parotid MALT-L show how IL-21-producing CD4^+^ Th cells represented the most expanded CD4^+^ population accounting for ~30% of the whole lesional CD4^+^ T-cells (figure 5A, B); in comparison Th17 cells, known to be involved in ELS formation,^33 34^ made up of less than 1% of cytokine-producing CD4^+^ T-cells within MALT-L. Interestingly, IL-21^+^ CD4^+^ T-cells frequently displayed concomitant production of IFN-γ but not IL-17, IL-10 or Granzyme-B in both labial SG and parotid MALT-L which clearly distinguish these cells from CD4^+^ T-cells infiltrating inflamed human tonsils (online supplementary figure S3).

Dimensionality reduction analysis (t-Distributed Stochastic Neighbor Embedding, t-SNE), revealed that IL-21 and IFN-γ-producing lesional T-cells in MALT-L fell within both ICOS^+^ and PD1^+^ CD4^+^ T-cells but displayed variable expression of CXCR5 (figure 5C). Accordingly, both ICOS (from ~70% to ~90%) and CXCR5 (from ~30% to ~60%) expression were enriched in IL-21-single producers compared to single IFN-γ or IL-21/IFN-γ-double producers (figure 5D), suggesting that *bona fide* CD4^+^CD25^-^Foxp3^-^CXCR5^+^ICOS^+^PD-1^+^ Tfh-cells account for most of lesional IL-21 production in parotid MALT-L. Of interest, the CXCR5^-^ population of CD4^+^PD-1^hi^ T-cells displayed a phenotype highly compatible with Tph-cells, with >95% ICOS expression and enriched in both IL-21 and IFN-γ production (figure 5E). Overall, these data support the hypothesis that the aberrant expansion of IL-21 and IL-21/IFN-γ-producing ICOS^+^PD-1^+^CD4^+^ T-cells, independently from CXCR5 expression, represents an important step in ELS maintenance and MALT-L development in the SG of patients with SS.

### Functional ICOS-blockade controls the release of IL-21 and other proinflammatory cytokines in SG organ cultures

**Figure 6:**
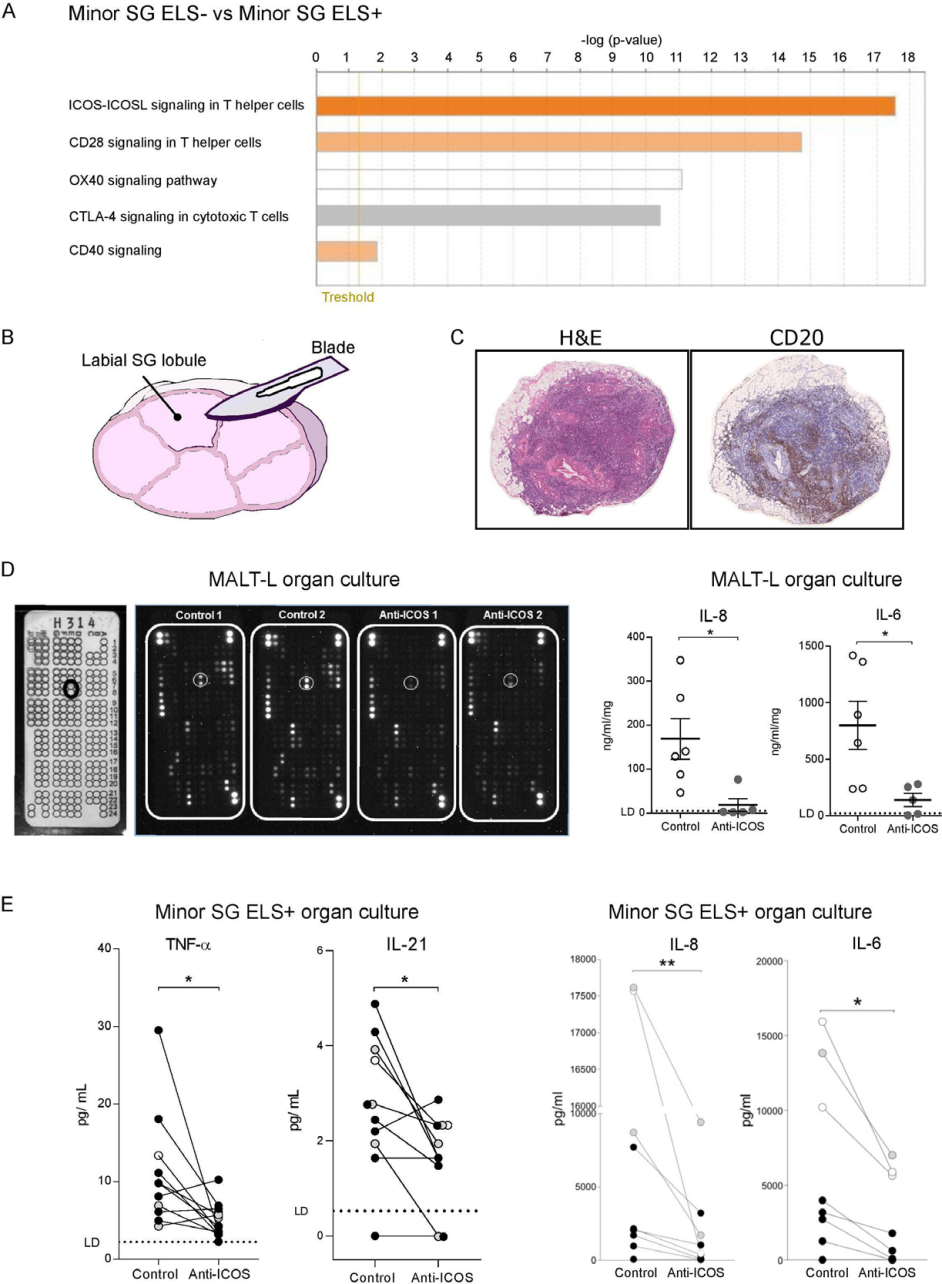
The blockade of ICOS-ICOS ligand signalling pathway reduces pro-inflammatory cytokines level in SG with ELS and MALT-lymphoma organ culture in SS. (A) Ingenuity Pathway Analysis (IPA) of microarray data obtained from the analysis of RNA extracted from ELS- and ELS+ SG. The orange-coloured bars (ICOS-ICOSL, CD28, CD40) show predicted pathway activation (with positive z-score), while the white bar (OX40 signalling pathway) indicates a z-score at or very close to 0 and the grey bar (CTLA4) pathways where no prediction can be made. (B) Schematic representation of a labial minor SG lobule cut longitudinally in half for the organ culture experiment. (C) representative histological images (H&E and IHC for CD20) of inflammatory infiltration in a parotid SG with B-cell non-Hodgkin MALT-lymphoma from a patient with SS. (D) Multiplex antibody array for cytokine analysis in the supernatant of a parotid MALT-lymphoma (n=1) organ culture, treated with an anti-ICOS blockade or its isotype control. The array template (white panel on the left) shows the coordinate reference of analytes with IL-6 spots highlighted (black circle). Black panels show the arrays incubated with organ culture supernatants treated with isotype control or anti-ICOS blockade with white circles around IL-6 spots. ELISA quantification of IL-8 and IL-6 in the supernatants of MALT-lymphoma organ culture, treated with an anti-ICOS blockade (grey dots) or its isotype control (white dots). Each dot represents a technical replicate. (E) Levels of TNF-α, IL-21, IL-8 and IL-6 detected in the supernatant of minor SG lobule organ culture, treated with an anti-ICOS blockade or its isotype control. The same colour dots represent minor SG lobules (from 2 to 8 lobules) from the same patient with SS (patients with SS, n=3). The lines link the two halves of the same lobules treated with the anti-ICOS blockade or its isotype control. Limit of detection (LD) for each cytokine is highlighted with a dashed line. ELISA quantification of IL-8 and IL-6 in the supernatants of minor SG lobules organ culture, treated with an anti-ICOS blockade or its isotype control, for cytokine levels that reach the Legendplex’ upper detection limit. Statistical analysis by Wilcoxon t-test. *p<0.05, **p<0.01. (F) All graphs represent mean±SEM. *P<0.05, **P<0.01, ***P<0.001, ****P<0.0001. ELS, ectopic lymphoid structure; ICOS, inducible T-cell costimulator; IFN-γ, interferon-γ; IHC, immunohistochemistry; IL-21, interleukin-21; MALT, mucosa-associated lymphoid tissue; NSCS, non-specific chronic sialoadenitis; PD-1, programmed cell death protein 1; SG, Salivary gland; SS, Sjogren’s syndrome; Tfh, T-follicular-helper.

Taken together, our data clearly indicate an expansion of ICOS^+^CD4^+^ T-cell subsets (both Tfh-like and Tph-like) in patients with SS with ELS and MALT-L. Additionally, hierarchical analysis of costimulatory pathways of microarray data identified ICOS-ICOS-L as the most upregulated pathway in SG with ELS (figure 6A), supporting a critical role for ICOS. In order to functionally investigate the importance of this pathway we set up organ cultures using either ELS+ labial SG (figure 6B) or parotid MALT-L tissue (figure 6C) incubated with a blocking, non-depleting anti-ICOS monoclonal antibody (mAb) or its isotype control. In parotid MALT-L tissue treated with the anti-ICOS mAb, a protein array screening assay on the organ culture supernatant showed down-modulation of several proteins including IL-8 and IL-6 as also confirmed by ELISA (figure 6D). Likewise, analysis of minor SG-organ culture supernatants showed that incubation with the anti-ICOS mAb reduced the levels of IL-21, TNF-α, IL-6 and IL-8 (figure 6E) in comparison to isotype control-treated glands.

Overall, these data confirm that the ICOS-ICOSL pathway is critical for the activation of important T-cell related proinflammatory pathways in both labial and parotid glands.

## Discussion

Here, we present a comprehensive analysis of peripheral and lesional CD4^+^ T-cells at different stages of SG immunopathology in a large cohort of patients with SS, a disease in which ectopic GC have been linked to the progression towards severe extraglandular manifestations and B-cell MALT-L.^4^ To start with, we reported the first high throughput transcriptomic profiling of ELS+ and ELS- SG tissues. Microarray analysis identified, using both unsupervised and supervised cluster analysis, a Tfh-cell signature, IL-21 and the ICOS-costimulatory pathway as the most upregulated gene clusters in the ELS+ vs ELS- subset of patients with SS. Using quantitative PCR and *in situ* hybridisation on SG tissue we confirmed that IL-21 mRNA was highly upregulated in SG with ectopic GCs and lesional Tfh-like cells. Strikingly, an aberrant expansion of infiltrating Tfh-like cells acquiring expression of the key transcription factor BCL6 was observed in parotid MALT-L, together with a disproportionate upregulation in IL-21 transcripts. Characterisation of T cell infiltration in control parotid was limited by the lack of matched histology samples with total RNA. These results are of great interest as MALT-L in SS arises from post-GC B cells with a marginal zone phenotype often bearing a RF+ autoreactive B cell receptor.^35^ Although extra follicular mechanisms are also likely to be involved in neoplastic B cell expansion, including B cell receptor cross-linking^36^ and aberrant NFkB activation^37^ via engagement of BAFF and Toll-like receptors^4^, the evidence of ongoing intratumour clonal diversification and aberrant somatic hypermutation suggest that ectopic GCs play an active role in supporting B cell lymphomagenesis.^22 38^ Deep phenotyping of lesional CD4^+^ T-cells in parotid MALT-L by flow cytometry revealed unexpected and highly novel results whereby we identified two main CD4^+^ T-cell subsets responsible for IL-21 production. One subset represented the *bona fide* Tfh-cells (Foxp3^-^CXCR5^+^ ICOS^+^PD1^+^) while the other, which accounted for around 50% of total IL-21 production in parotid MALT-L, shared striking similarities with the recently described CXCR5^-^PD1^hi^ICOS^+^ pathogenic Tph-cells.^19^ Although these results should be interpreted with caution, as FACS analysis was performed on one MALT-L sample, together with transcriptomic and histology data on a larger cohort, our results provide the first evidence that this subset may be directly implicated in MALT B-cell-lymphomagenesis being uniquely able to provide proliferative B-cell signals at extrafollicular sites. Additionally, both Tfh and Tph-like cells in parotid MALT-L and ELS+ SG displayed heterogeneity in cytokine production with expanded populations of single IL-21, single IFN-γ and double IL-21/IFN-γ producers; it is unclear whether these represent distinct or evolutionary subsets driven by the local inflammatory milieu but our work prompts further investigation into our understanding of the heterogeneity and pathogenic properties of the expanding family of Tfh-like cells.^39^

Due to the heterogeneity in marker combinations used to identify peripheral Tfh-cells in patients with SS in previous works,^28 40–44^ we performed a comprehensive analysis of Tfh-like cell subsets in peripheral blood matched with SG on the basis of the expression of CXCR5, ICOS and PD-1 coupled with intracellular cytokine analysis. Our work indicated that circulating CXCR5^+^ICOS^+^PD-1^+^ Tfh-cells, which represent a partially differentiated state compared with Tfh in secondary lymphoid organs, but are able to maintain B-cell differentiating functions^45^ are significantly expanded in ELS+ patients. Notably, the expansion of IL-21 producing CXCR5^+^ICOS^+^PD-1^+^ Tfh subsets positively correlated with SG focus score and these Tfh-cells were selectively enriched in patients with ELS in the SG biopsies suggesting that IL-21^+^ Tfh-cells may represent a useful blood biomarker of SG immunopathology. Additionally, we further expanded on previous works by showing how circulating CXCR5^+^ICOS^+^PD-1^+^ Tfh subsets producing high levels of both IL-21 and IFN-γ (including the detection of double IL-21/IFN-γ producers) are selectively enriched in patients with SS with anti-Ro/SSA and anti-La/SSB autoantibodies, positively correlated with IgG levels and negatively with complement C4. Overall these data support the notion that circulating Tfh in patients with SS play an active role in the autoimmune B-cell dysregulation typical of patients with SS as previously suggested.^41 42^ These data also link well with previous work showing that an over expansion of Tfh-cells is associated with a dysregulation of B-cell dynamics^44^ and the production of autoantibodies^46^ and that an expansion of Tfh-cells might lower the selection pressure on GC B-cells, leading to the emergence of autoreactive B-cells.^47^

Finally, in addition to identifying IL-21 and ICOS as key pro-inflammatory and co-stimulatory signals promoting autoreactive B-cell activation and lymphoma progression in patients with SS, we also provide proof-of-concept functional evidence in SG organ cultures that candidates blocking the ICOS-pathway as a novel therapeutic option in SS. Here we report that an anti-ICOS non-depleting blocking antibody incubated with either ELS+ SG biopsies or parotid MALT-L induced a drastic downregulation of IL-21 and other key proinflammatory cytokines. These data are in line with the evidence that ICOS is required for the reciprocal activation of T-cell to B-cells^48^ and that IL-21 expression by Tfh requires the interaction with ICOS-L (which can also be expressed by SG epithelial cells).^49^ Our results are highly relevant to the recently completed phase IIa clinical trial with an anti-ICOSL mAb in primary SS (NCT02334306) and will help interpreting the clinical efficacy of targeting the ICOS/ICOSL costimulatory pathway in SS by prompting the use of patient stratification on the basis of lesional and/or peripheral Tfh-cell signatures to achieve maximum clinical efficacy.
