# Supplementary material for: Unique expansion of IL-21+ Tfh and Tph cells under control of ICOS identifies Sjögren’s syndrome with ectopic germinal centres and MALT lymphoma
Source: Ann Rheum Dis. 2020 Sep 22;79(12):1588–99. doi: 10.1136/annrheumdis-2020-217646 (PMC7677495; doi:10.1136/annrheumdis-2020-217646)
Supplement: Supplementary data [file annrheumdis-2020-217646supp001.pdf]

Supplementary Material

Supplementary Figure 1

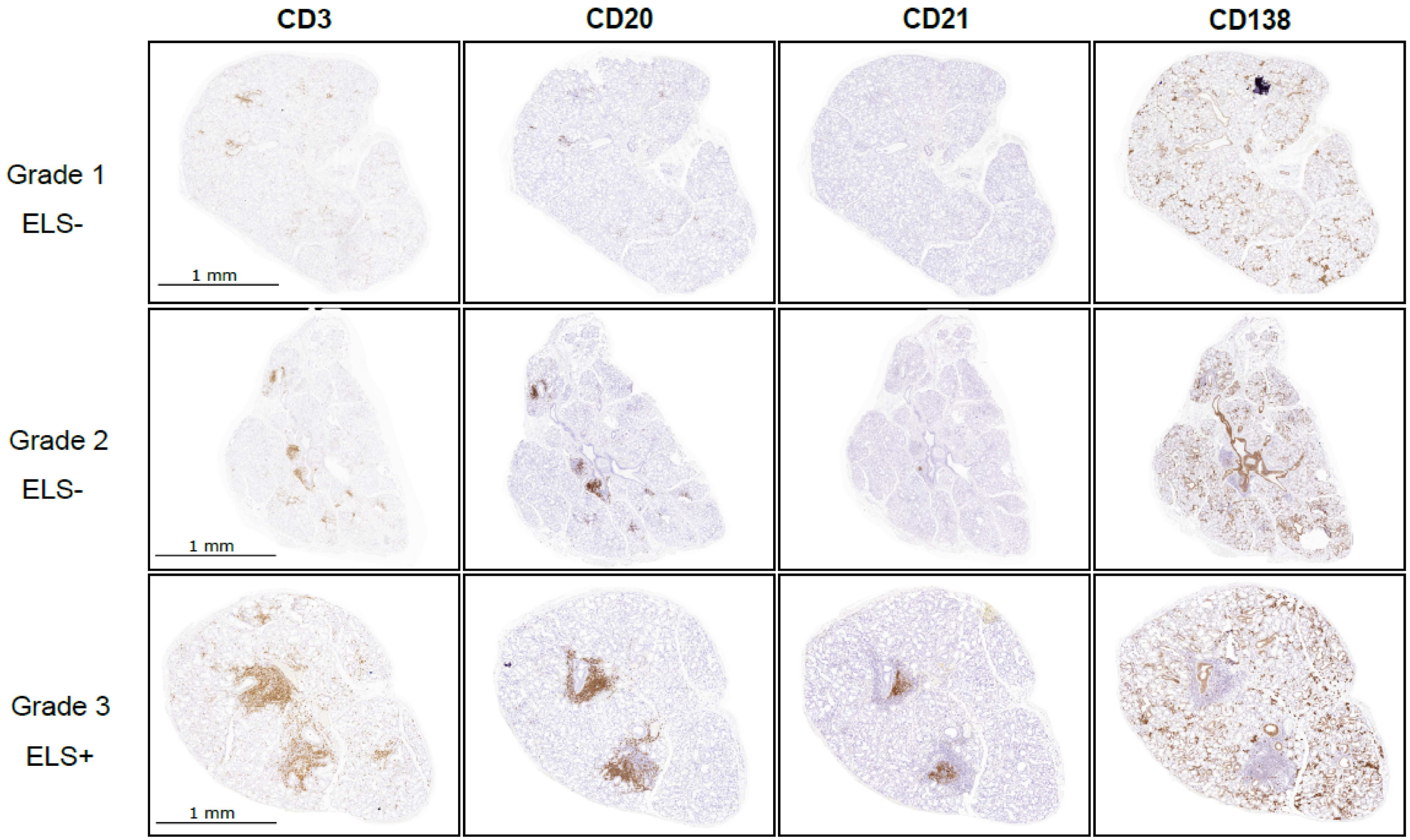

Figure S1. Histological grading and ELS classification of labial SG

The immune cell type and the degree of infiltration of SG biopsies were assessed by IHC staining for T cells (CD3), B cells (CD20), the presence of follicular dendritic cell (FDC) network (CD21) and plasma cells (CD138). The degree of infiltration of each immune cell type was scored using a semi-quantitative score (0 to 3) and further classified in ELS+ or ELS-. The histological identification of ELS in the labial SG biopsies is defined as at least one infiltrate with clear B/T cell segregation and presence of FDC, suggestive of germinal center presence.

Supplementary Figure 2

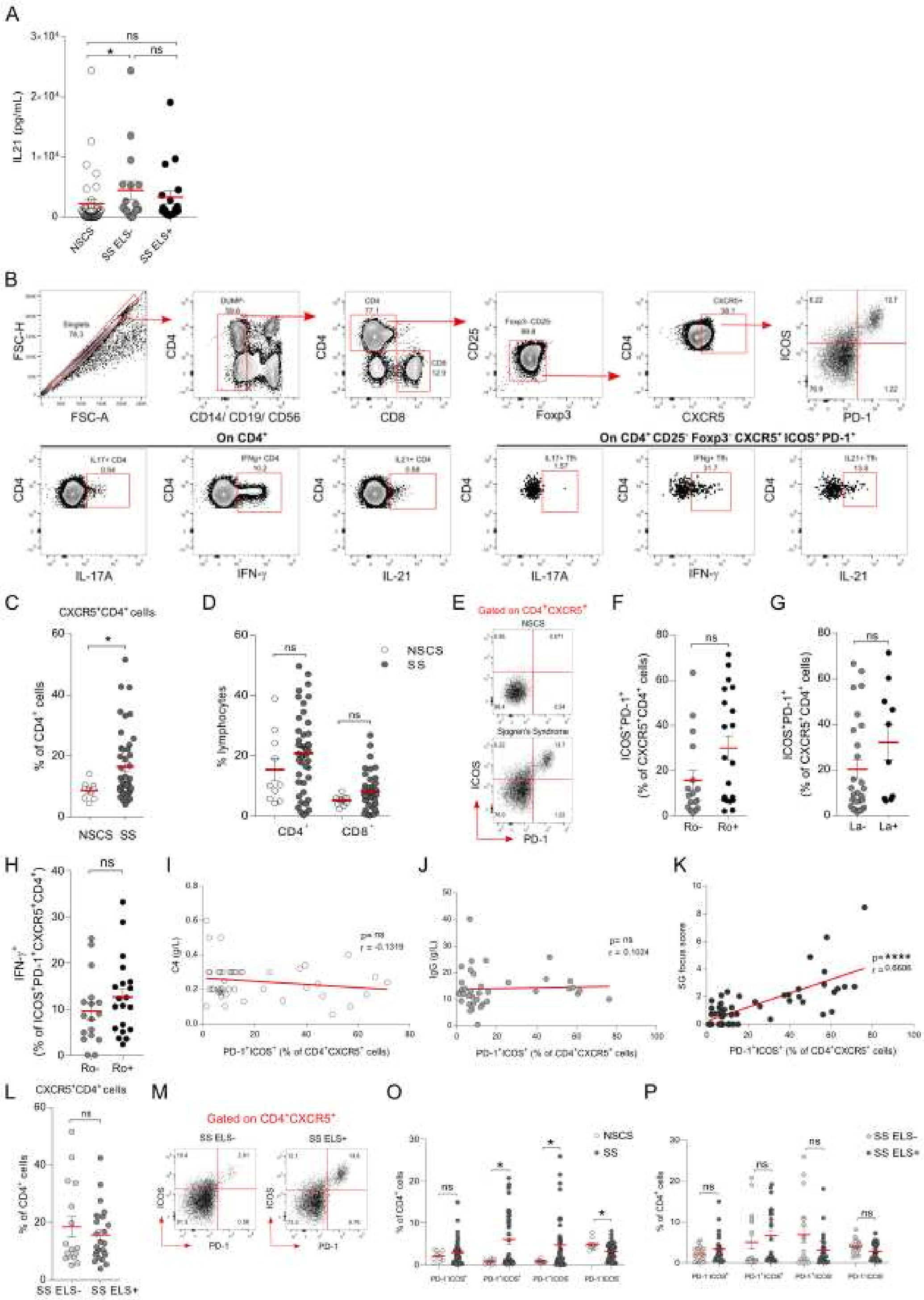

Figure S2. Frequency and cytokines production of circulating Tfh cells, in peripheral blood of SS and NSCS patients.

(**A**) ELISA quantification of IL-21 serum level (pg/mL) in NSCS (n=37) and SS cohort segregated for ELS presence, ELS- (n=18), ELS+ (n=18). Statistical analysis by Kruskal-Wallis-test with Dunn's post-test correction for multiple comparisons. (**B**) Flow-cytometry gating strategy for circulating T helper compartment focusing on Tfh cells (identified as $CD4^+CD25^-Foxp3^-CXCR5^+ICOS^+PD\text{-}1^+$) and their cytokines production. Regulatory markers CD25 and Foxp3 were included in the gating strategy to exclude Treg ($CD4^+CD25^+Foxp3^+$) and T-follicular regulatory cells ($CD4^+CD25^+Foxp3^+CXCR5^+ICOS^+PD\text{-}1^+$). FMO have been used to define threshold positivity. (**C**) $CXCR5^+CD4^+$ cell frequency, as percentage of $CD4^+$ T-cells, based on flow-cytometry analysis of PBMC from NSCS (n=10) and SS (n=42). (**D**) Frequency of $CD4^+$ and $CD8^+$ T cells as percentage of lymphocytes gate, identified on the basis of physical parameters (SSC-A and FSC-A), based on flow-cytometry analysis of PBMC from NSCS (white dots, n=10) and SS (dark grey dots, n=42). (**E**) Representative flow-cytometry dot plots for Tfh-cell subsets identified on the basis of ICOS and PD-1 expression. Frequency of $PD\text{-}1^+ICOS^+$ Tfh cells, as percentage of $CXCR5^+CD4^+$ cells in SS cohort segregated on basis of positivity for Ro (**F**) and La (**G**) auto-antibodies. (**H**) Distribution of IFN-γ producing $ICOS^+PD\text{-}1^+CXCR5^+CD4^+$ cells segregated on basis of positivity for Ro auto-antibodies. Spearman correlation analysis between frequency of $PD\text{-}1^+ICOS^+$ Tfh cells, as percentage of $CXCR5^+CD4^+$ cells, and C4 (**I**), IgG (**J**) and SG focus score (**K**). (**L**) Frequency of $CXCR5^+CD4^+$ cells, as percentage of $CD4^+$ cells in SS cohort segregated on basis of ELS presence in the SG biopsies. (**M**) Representative flow-cytometry dot plots for Tfh-cells subsets identified on the basis of ICOS and PD-1 expression in SS cohort segregated for ELS presence. (**O**) Frequencies distribution of Tfh-cell subsets, identified on the basis of ICOS and PD-1 as percentage of $CD4^+$ cells, in NSCS (white dots, n=10) and SS (dark

grey dots, n=42) and (**P**) within SS cohort segregated for ELS presence [ELS-, light grey dots (n=17), ELS+, dark grey dots (n=25)]. All graphs represent mean ± SEM. *p<0.05, **p<0.01, ***p<0.001, ****p<0.0001. Abbreviations: Non Specific Chronic Sialoadenitis (NSCS), Sjogren's syndrome (SS), Ectopic Lymphoid Structure (ELS).

Supplementary Figure 3

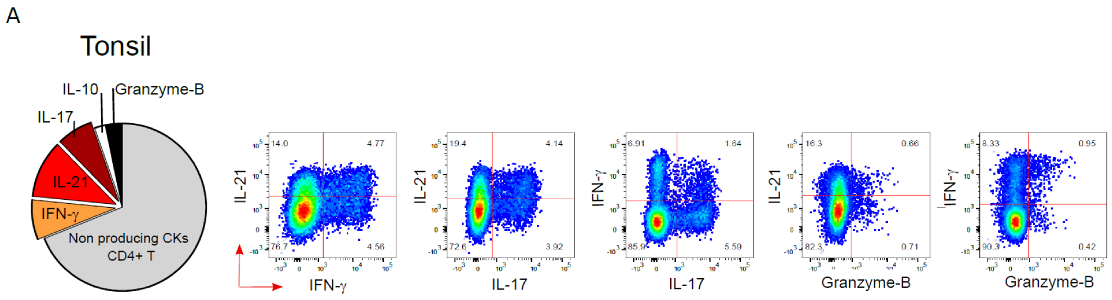

Figure S3. Cytokines production by T helper ($CD4^+$) compartment in human tonsil. The pie chart show the average frequencies of the main cytokines produced by T helper cells, as percentage of $CD4^+$ T cells, in human tonsil (n=1). Representative flow-cytometry dot plots showing the cytokines production, gated on $CD4^+$ T cells.

Supplementary Figure 4

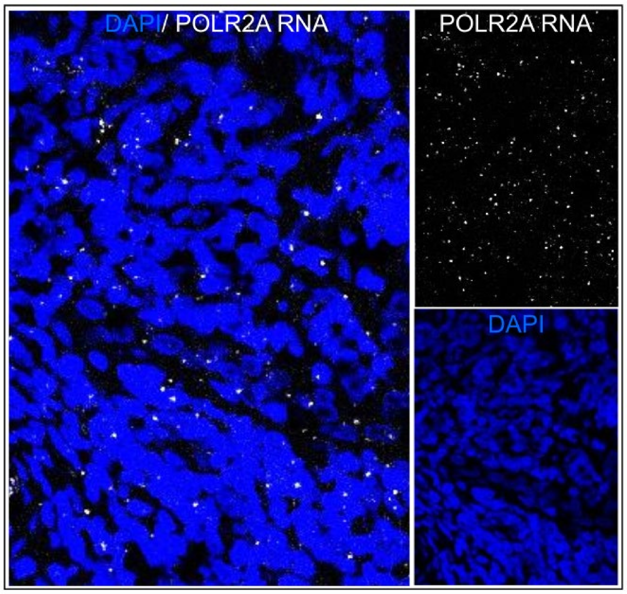

Figure S4. Positive control for IL-21 RNA fluorescence in situ hybridization (FISH). Sjogren's syndrome minor salivary gland section stained with #310451 RNAscope Positive Cont Probe Hs-POLR2A, as positive control for IL-21 RNA (FISH).

Table S1: Antibodies used for immunohistochemistry (IHC) and immunofluorescence (IF) on formalin fixed paraffin embedded (FFPE) sections

Antigen	Conjugation	Clone	Species	Manufacturer	Catalogue	Application	Dilution
CD20	Unconjugated	L-26	Mouse IgG2a	DAKO	M0755	FFPE IHC	1:20
CD3	Unconjugated	F.7.2.38	Mouse IgG1	DAKO	M7254	FFPE IHC	1:50
CD138	Unconjugated	M115	Mouse IgG1	DAKO	M7228	FFPE IHC	1:50
CD21	Unconjugated	1F8	Mouse IgG1	DAKO	M0784	FFPE IHC	1:30
CD4	Unconjugated	4B12	Mouse	DAKO	M7310	FFPE IHC	1:50
CD45RO	Unconjugated	UCHL1	Mouse	DAKO	M0742	FFPE IHC	1:70
ICOS/ CD278	Unconjugated	SP98	Rabbit	Spring Bioscence	M3982	FFPE IHC	1:50
PD1	Unconjugated	NAT	Mouse	Hybridome supernatant	/	FFPE IHC	1:10
BCL6	Unconjugated	PG-B6p	Mouse	DAKO	M7211	FFPE IHC	1:50
CD68	Unconjugated	KP1	Mouse	DAKO	M0814	FFPE IHC	1:50
Mouse IgG1	Alexa488	Polyclonal	Goat	Invitrogen	A-21121	FFPE IF	1:200
Mouse IgG2a	Alexa488/ Alexa555/ Alexa647	Polyclonal	Goat	Invitrogen	A-21131/ A-21137/ A-21241	FFPE IF	1:200

Table S2: Antibodies used for FACS staining

Antigen	Conjugation	Clone	Species	Manufacturer	Dilution
CD14	BV510	M5E2	Mouse IgG2a, κ	Biolegend	1:40
CD19	BV510	HIB19	Mouse IgG1, κ	Biolegend	1:100
CD56	BV510	HCD56	Mouse IgG1, κ	Biolegend	1:50
CD4	PEDazzle 594	RPA-T4	Mouse IgG1, κ	Biolegend	1:300
CD8	APC Cy7	SK1	Mouse IgG1, κ	Biolegend	1:40
CXCR5	BV605	J252D4	Mouse IgG1, κ	Biolegend	1:160
ICOS	PECy7	C398.4A	Armenian Hamster IgG	Biolegend	1:400
PD-1	PerCp/Cy5.5	EH12.2H7	Mouse IgG1, κ	Biolegend	1:80
CD25	BV650	BC96	Mouse IgG1, κ	Biolegend	1:80
Foxp3	PE	150D	Mouse IgG1, κ	Biolegend	1:20
IL-17A	BV711	BL168	Mouse IgG1, κ	Biolegend	1:20
IFN-γ	BV785	4S.B3	Mouse IgG1, κ	Biolegend	1:20
IL-21	AlexaFluor 647	3A3-N2	Mouse IgG1, κ	Biolegend	1:40
Granzyme-B	Pacific blue	GB11	Mouse IgG1, κ	Biolegend	1:20
IL-10	AlexaFluor 488	JES3-9D7	Rat IgG1, κ	Biolegend	1:20

Table S3: Genes used for gene signature in Gene Set Variation Analysis

Gene signature	Genes
**Bcell**	CD22, FCRLA, MS4A1, VPREB3, TCL1A, EBF1, FCRL1, BANK1
**PlasmaCell**	IGHA1, TNFRSF17, IGKV4-1, IGKC, IGJ
**Total_CD19+**	IGHM, LOC283663, BANK1, MS4A1, FCRLA, FAM129C, CD19, CD22, EBF1,IGHG1, VPREB3, TCL1A
**IFN21**	EPSTI1, HERC5, IFI27, IFI44, IFI44L, IFI6, IFIT1, IFIT3, ISG15, LAMP3, LY6E, MX1, OAS1, OAS2, OAS3, PLSCR1, RSAD2, RTP4, SIGLEC1, USP18, SPATS2L
**CD4_T_Cell**	RCAN3, PLEKHB1, BCL11B, GLTSCR2, RPL22, BAG3, RPL3P7, STMN3, RPL3, LTBP3, FAM102A, LOC439949, TCF7, GPRASP1, TRAC, SELM, RPL10A, NMT2
**T_EFFECTOR**	CD8A, CXCL10, CXCL9, EOMES, GZMA, GZMB, IFNG, TBX21
**TH1**	CXCL10, CXCL9, IFNG, IL12A, TBX21
**TH2**	GATA3, IL13, IL4, IL4R
**Tfh_markers**	CXCR5, ICOS, PDCD1, SH2D1A
**T_REG**	CCR7, FOXP3, IL10, IL2RA, ITGB2, TFRC, TGFB1
**Total_CD8**	CD8A, CD8B, KLRK1, LCK, CD2, ARL4C, ZAP70, IL32, ZNF827, ITK, SLC38A1, C6orf190, IL23A
**MONOCYTES**	CPVL, ASGR1, CLEC10A, RASSF4, PEA15, TGFBI, LY86, ASGR2, DPYSL2, CCDC88A, MYCL1, SLC46A2, PLXNB2, CCR2, VCAN, C4orf18, CTNND1, CD86, HLA-DMA, PID1

Table S4: TaqMan probes used for real-time PCR experiment

Gene name	Producer	Species	Cat. No.	Fluorophore
AID	Applied Biosystems	Human	Hs00221068_m1	FAM
BAFF	Applied Biosystems	Human	Hs00198106_m1	FAM
IL21	Applied Biosystems	Human	Hs00222327_m1	FAM
IL-21R	Applied Biosystems	Human	Hs00222310_m1	FAM
LTβ	Applied Biosystems	Human	Hs00242737_m1	FAM
Pax 5	Applied Biosystems	Human	Hs00172003_m1	FAM
Prdm (Blimp1)	Applied Biosystems	Human	Hs00153357_m1	FAM
CXCL13	Applied Biosystems	Human	Hs00757930_m1	FAM
18S	Applied Biosystems	Human	Hs99999901_s1	FAM
β-actin	Applied Biosystems	Human	Hs99999903_m1	FAM
